# Navigating a Curious Case of Unilateral Keratouveitis in Pakistan

**DOI:** 10.7759/cureus.12631

**Published:** 2021-01-11

**Authors:** Alvina Karam, Bakht D Khan, Abuzar Siraj, Bakht S Khan

**Affiliations:** 1 Surgery, Khyber Medical College, Peshawar, PAK; 2 Ophthalmology, Khyber Medical College, Peshawar, PAK; 3 Medicine, Hayatabad Medical Complex, Peshawar, PAK; 4 Ophthalmology, Khyber Teaching Hospital, Peshawar, PAK

**Keywords:** keratitis, keratouveitis, corneal ulcer, bacterial

## Abstract

Keratouveitis is a rare but potentially dangerous condition of the eye that requires prompt management otherwise it may result in severe visual impairment. We report a challenging case of unilateral keratouveitis.

A 40-year-old man presented with right eye erythema, epiphora, decreased vision, and severe pain from one month after failure to antibiotics treatment. Slit-lamp examination showed circumcorneal injection of conjunctiva, a central corneal ulcer, hypopyon and cells in the anterior chamber (AC). The culture and sensitivity of ocular tissue showed no growth of organisms. Intravitreal antibiotics (vancomycin and ceftazidime) along with anterior chamber tap led to resolution of the inflammation. The corneal ulcer was markedly reduced and vision was improved.

This case emphasizes two important points. First, how to approach a case of keratouveitis of uncertain etiology, in a less resourceful situation. Second, how to treat unilateral keratouveitis with intravitreal antibiotics and anterior chamber/vitreous tap.

## Introduction

Keratouveitis refers to inflammation of the cornea and the uvea, which consists of the iris, ciliary body, and choroid. Ocular signs and symptoms may include ocular pain, dry or red eyes, foreign-body sensation, conjunctival erythema, visual changes, and even complete visual loss. The etiologies of these ocular manifestations are allergic, infectious, autoimmune, and idiopathic causes. A complete ophthalmic examination encompasses external inspection, visual acuity, ocular motility, pupillary reaction, and ophthalmoscopy. Slit-lamp examination with fluorescein dye is necessary for diagnosis and prognosis of lesion. Histopathological evaluation of ocular tissues is also significant when it comes to differentiating between infectious and autoimmune etiologies. It helps in establishing a definitive diagnosis and targeted management of the patient based upon underlying etiology.

The causative organisms include herpes simplex virus (HSV) keratitis, mycotic keratitis, acanthamoeba keratitis, and bacterial keratitis. Essential laboratory tests for patients include culture and smears to narrow down an infectious etiology. Bacterial keratitis presents with features of ulcerative keratitis with infiltrates and suppuration. Moreover, the diagnosis of HSV keratouveitis is made in patients with a known history of HSV keratitis confirmed by typical dendritic epithelial defects and the use of polymerase chain reaction (PCR) [1]. 

Bacterial infections are still preponderant and are found in 80% of patients with keratitis [2]. According to another article, *Pseudomonas aeruginosa* is among the most common causes of bacterial keratouveitis [3]. The incidence rates for bacterial keratitis ranges from approximately 2.2-4.1/10,000 per year for daily-wear soft contact lenses [4]. In our case, the etiology was still uncertain. 

We have presented a complicated case of unilateral keratouveitis successfully treated with a combination of anterior chamber/vitreous tap and intravitreal antibiotics leading to near-complete resolution of the corneal ulcer. The manifestations of the disease were quite similar to and suggestive of infectious cause but the negative culture reports ruled out any bacterial origin. However, in our setup, where resources are scarce, it was not possible to ascertain the etiology.

Informed consent was obtained from the patient for this study. 

## Case presentation

A 40-year-old man presented to us with a one-month history of pain and watery discharge of his right eye. The patient also complained of right eye vision loss and right sided headache for the last couple of days. He used topical antibiotics and steroids initially for a corneal ulcer which led to further deterioration of his vision. The rest of his past medical history was not significant.

**Figure 1:**
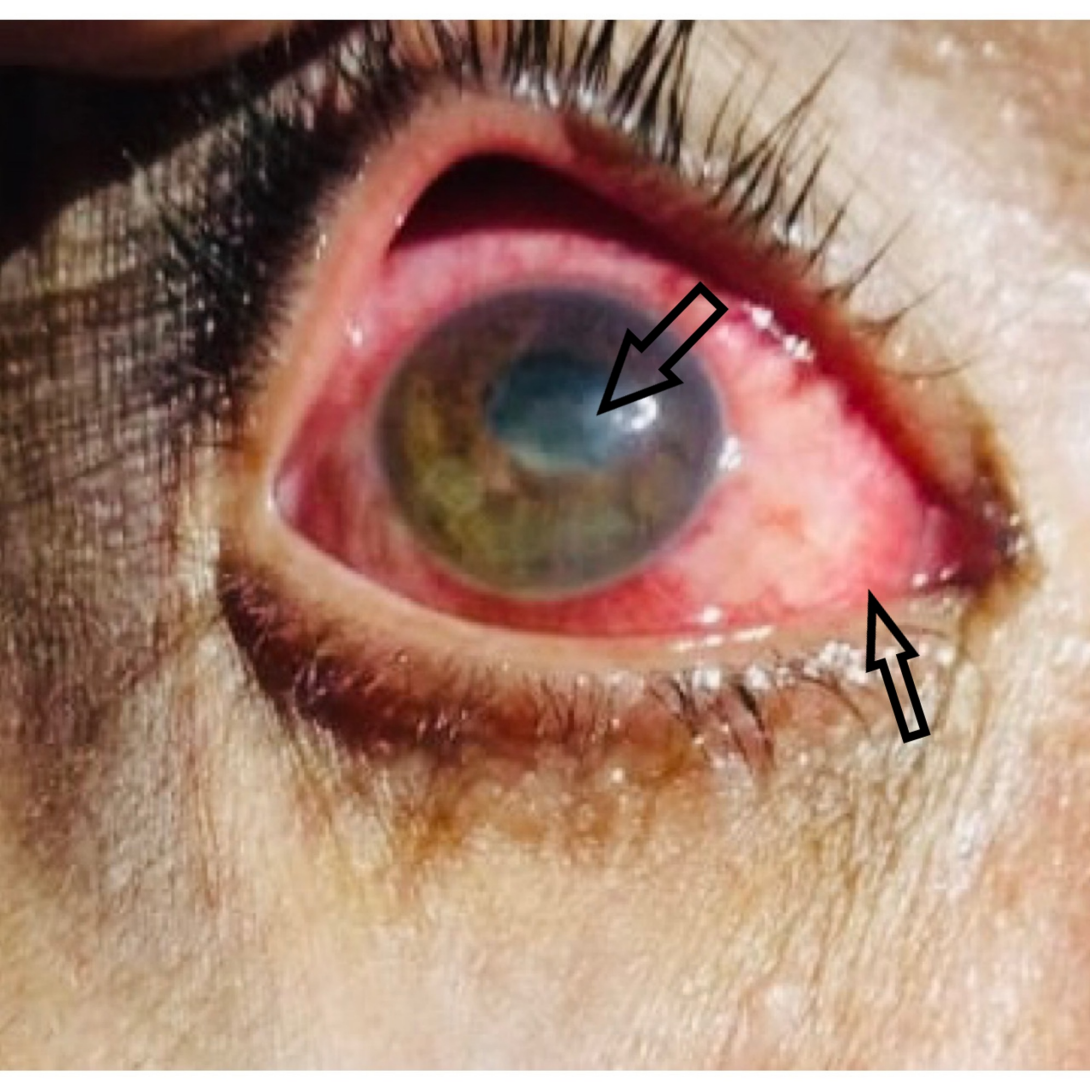
Gross view of the Right eye showing congestion with a central opacity

**Figure 2:**
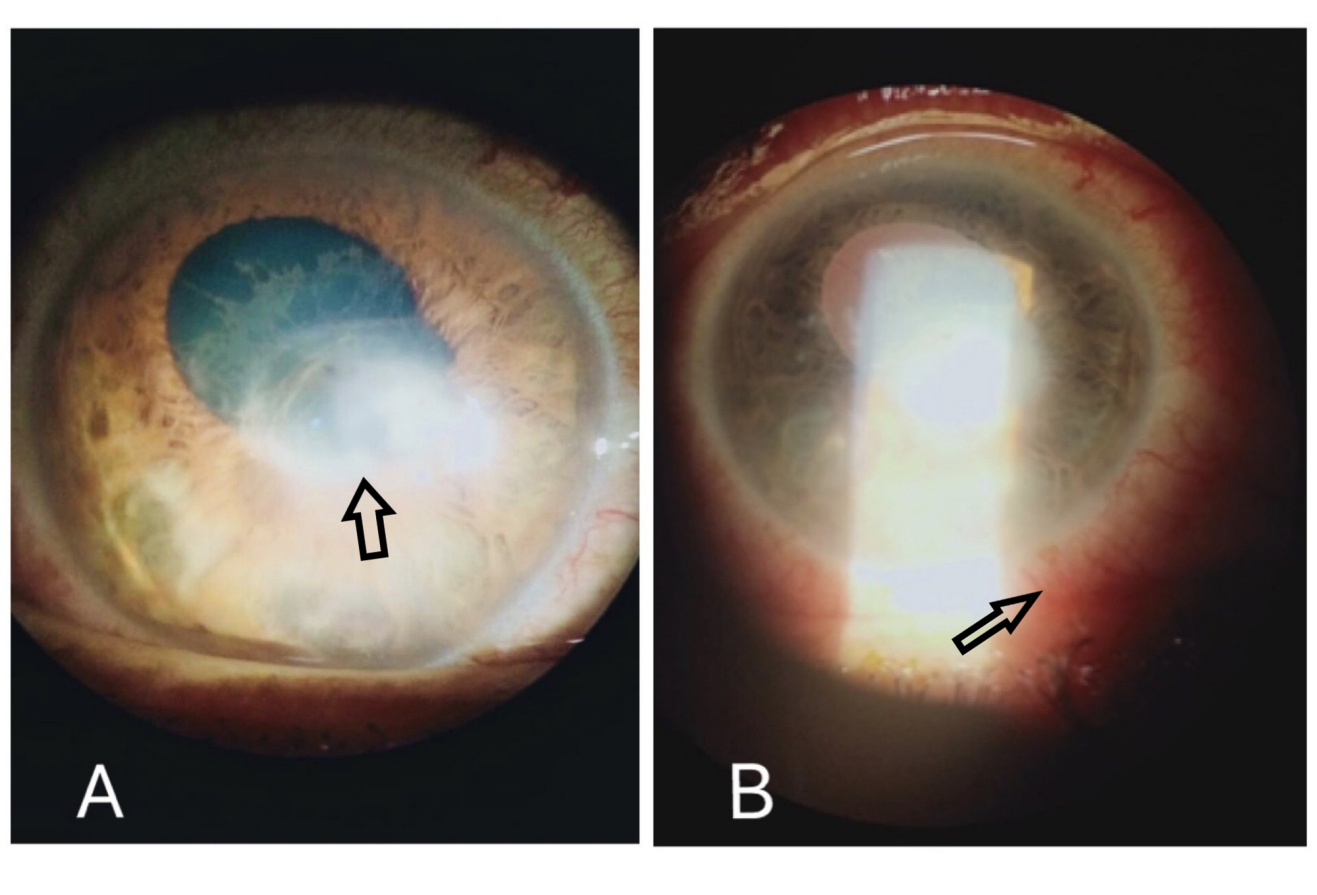
Slit lamp Images of the right eye. Slit Lamp Examination showing central corneal ulcer, hypopyon, cells in the anterior chamber (A) and circumcorneal injection of conjunctiva (B),

**Figure 3:**
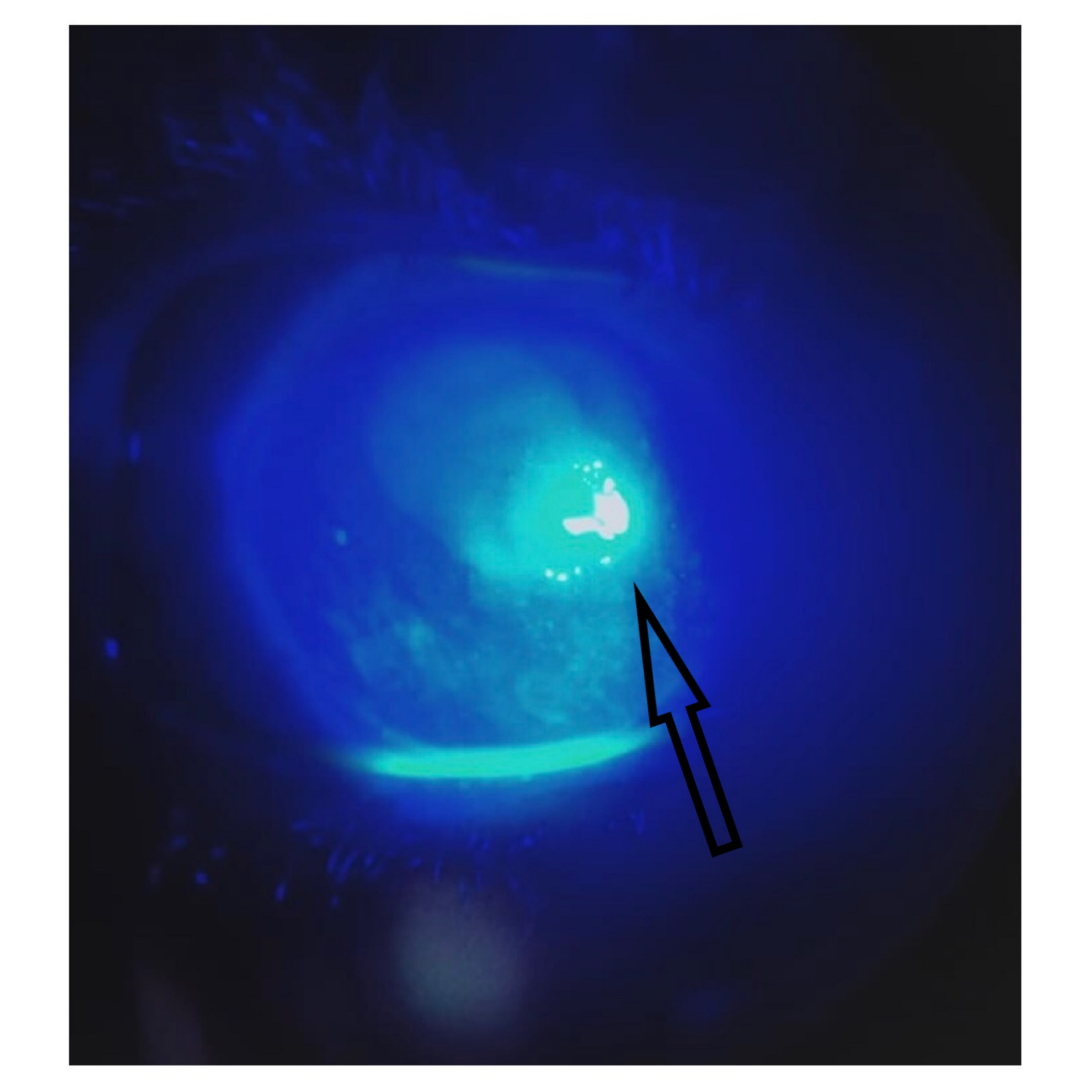
Fluorescein staining of the cornea

Examination of the right eye showed tearing and congestion with a central opacity (Figure 1). Visual acuity at the time of admission was light perception in the right eye and 6/6 in the left. Ocular movements were normal and without pain. The right eye was non-reactive to direct and consensual light reflex. The intraocular pressure was 12 mmHg in the right eye. Slit-lamp examination of the right eye showed circumcorneal injection of conjunctiva, central corneal ulcer, hypopyon, and cells in the anterior chamber (AC) (Figure 2). The cornea was covered by membrane and it stained lightly with fluorescein (Figure 3).

The systemic examination was unremarkable. There were no signs and symptoms suggestive of other associated diseases like HSV, rheumatoid arthritis, or inflammatory bowel disease. He denied having fever, flu-like symptoms, joint pains, or any changes in his bowel habits. 

The corneal scrape was sent immediately for culture and sensitivity. Surprisingly, the corneal cultures obtained no growth. The complete blood count was also normal. The patient denied having further investigations done due to scarcity of funds. 

The patient was diagnosed as a case of keratouveitis and started promptly on corticosteroids (dexamethasone 0.1% ophthalmic solution one drop every four hours), antibiotics (moxifloxacin oral 400 mg and 5 mg/ml ophthalmic solution one drop every two hours, moxifloxacin ointment), cyclopentolate HCl (1% ophthalmic solution three times a day), natamycin (ophthalmic solution one drop every two hours).

During the following days, the pain partially subsided and mild improvement in vision was seen. Visual acuity was 3/60 in the right eye and 6/6 in the left. Slit-lamp examination showed ulcerated nodules, dilated blood vessels, and synechiae in the iris. No AC cells were seen. Subsequently, azithromycin 250 mg was added to the treatment regimen.

Despite 10 days of treatment, there was mild pain in the right eye, and the hypopyon and corneal ulcer persisted. Visual acuity decreased, once again, to light perception in the right eye. Slit lamp examination showed collapsed AC and subconjunctival hemorrhage. The intraocular pressure (IOP) increased to 42mm Hg for which IV mannitol (300 ml) was advised.

At this stage, a diagnostic and therapeutic vitreous and anterior chamber tap was performed under general anesthesia. The vitreous tap was sent for culture and sensitivity but obtained no growth in 24 hours. The patient was then administered intravitreal antibiotics vancomycin 2 mg/0.1 ml and ceftazidime 2 mg/0.1 ml.

With continued therapy, the patient showed significant improvement. By the fourth week of treatment with intravitreal antibiotics, there was near complete resolution of the corneal ulcer and ocular inflammation. A marked clinical response was seen with three doses of intravitreal antibiotics and anterior chamber tap. The visual acuity increased to 3/60 in the right eye. The patient was then lost to follow-up.

## Discussion

Keratouveitis results in inflammatory destruction of the cornea and the uvea. Thus, the timely diagnosis of keratouveitis requires a thorough history, physical and ocular examination, and appropriate laboratory testing. Among the different causes of infectious keratouveitis are HSV, bacterial, fungal and acanthamoeba including non infectious causes which are scleritis and peripheral ulcerative keratitis. A rare cause of keratouveitis is endogenous *Listeria monocytogenes* endophthalmitis [5]. The mean annual incidence and prevalence rates (per 100,000 population in Finland) of idiopathic anterior uveitis were 17.1 and 48.5, respectively [6]. According to another study, uveitis was idiopathic in 44.6% of the population [7].

A careful history can help to narrow the likely causes of keratouveitis, as recent trauma, contact lens wear, a mucoid discharge, and past history of HSV may indicate allergic or infectious etiology. HSV keratitis is the leading cause of corneal blindness in developed countries [8]. The recurrent nature of the HSV keratitis may result in persistent corneal epithelial defects and development of corneal scarring [9]. The use of PCR is helpful in diagnosing HSV keratouveitis. The peculiar dendritic epithelial defects are suggestive of HSV.

Moreover, the appropriate diagnostic criteria are based on the slit-lamp examination findings, including culture and sensitivity reports of ocular tissue for identification of bacterial etiologies. Epithelial defects and injuries are key predisposing factors making the eye susceptible to ocular pathogens. The most common agents responsible for keratitis include *Staphylococcus aureus* and *Pseudomonas aeruginosa* [10]. Bacterial keratouveitis presents with a characteristic stromal ulceration and infiltrates.

In our patient, the clinical manifestations and slit-lamp findings were quite similar to infectious causes like bacterial and fungal but the negative cultures ruled out an infectious etiology. The past medical history was also negative. On systematic examination, the patient had no other signs and symptoms apart from the ocular findings. The complete blood count results were normal. We could have done HSV PCR but the patient denied to undergo further investigations, which indeed made it difficult to ascertain the etiology and manage the patient in scarce resources. 

Based on our diagnostic workup, there was some unknown etiology of the keratouveitis in our patient. There was no obvious cause for the ocular inflammation. We had to take immediate steps in order to prevent our patient’s vision from deteriorating. A combination of corticosteroids and antibacterial ophthalmic solutions were prescribed to the patient.

However, an interesting argument that can be made is that the patient had already been using antibiotics before presenting to us and the negative cultures could have been a result of that. But, if the offending agent was indeed of bacterial etiology and was sensitive to the antibiotic then it would have resulted in clinical improvement, which was not the case in our patient. Our management was centered around broadening the spectrum of antibiotic coverage to counter not only bacterial agents, but also fungal. However, no improvement in vision and inflammation was seen and the corneal ulcer persisted. Worryingly, two days later the intraocular pressure also rose. 

To our knowledge, there is no standard treatment for keratouveitis of unknown etiology. AC and vitreous taps were performed for diagnostic and therapeutic purposes. Vitreous needle tap is deemed to be a safe clinical procedure with a high success rate. AC paracentesis is also a valuable procedure in the management of uveitis, particularly in diagnosing infective causes. It was also intended to use therapeutically to lower intraocular pressure. Final resolution in the symptoms was seen with three doses intravitreal antibiotics (vancomycin 2 mg/0.1 ml and ceftazidime 2 mg/0.1 ml).

## Conclusions

The case we are presenting is unique to us due to the manner in which it progressed and manifested, carrying a lot of the characteristics of classic bacterial keratitis and uveitis. However, our investigations pointed toward no such offending agent. We utilized all of our available resources to give the patient a blend of treatment which we hoped would result in the optimum result. Our initial treatment failed to make any significant impact. However, when combining different facets of keratouveitis treatments, the patient’s vision was restored. The role of intravitreal antibiotics and vitreous tap cannot be underestimated in this case that helped alleviate the symptoms. However, it is rarely if ever the first option to treat such a case. We hope that further research will be done for recommendations and guidelines in managing a patient with keratitis, especially when the diagnostic resources are limited and the investigations point in no general direction. 
